# The rapid and visual detection of methicillin-susceptible and methicillin-resistant *Staphylococcus aureus* using multiplex loop-mediated isothermal amplification linked to a nanoparticle-based lateral flow biosensor

**DOI:** 10.1186/s13756-020-00774-x

**Published:** 2020-07-17

**Authors:** Xu Chen, Kai Ma, Xu Yi, Lijuan Xiong, Yu Wang, Shijun Li

**Affiliations:** 1 Central Laboratory of the Second Affiliated Hospital, Guizhou University of Traditional Chinese Medicine, Guiyang, Guizhou 550003 P.R. China; 2 The Second Clinical College, Guizhou University of Traditional Chinese Medicine, Guiyang, Guizhou 550003 P.R. China; 3 Laboratory of Bacterial Infectious Disease of Experimental Center, Guizhou Provincial Centre for Disease Control and Prevention, 73 Bageyan Road, Guiyang, Guizhou 550004 P.R. China; 4 Department of Clinical Laboratory Centre, The First People’s Hospital of Guiyang, Guiyang, Guizhou 55004 P.R. China

**Keywords:** *Staphylococcus aureus*, MRSA, Limit of detection, Loop-mediated isothermal amplification, Lateral flow biosensor

## Abstract

**Background:**

*Staphylococcus aureus* (*S. aureus*), including methicillin-susceptible *S. aureus* (MSSA) and methicillin-resistant *S. aureus* (MRSA), is an eminent human pathogen that can colonize the human host and cause severe life-threatening infections. The development of a reliable, simple and rapid assay for detecting *S. aureus* and identifying MRSA is important for diagnosis and follow-up treatment.

**Methods:**

A novel molecular diagnosis technique, named multiplex loop-mediated isothermal amplification linked to a nanoparticle-based lateral flow biosensor (m-LAMP-LFB), was applied to detect all *S. aureus* species and identify MRSA. Two sets of primers were designed based on the *femA* gene (*S. aureus*-specific gene) and the *mecA* gene (encoding penicillin-binding protein 2a), and the multiple-LAMP products were analyzed using LFB. The m-LAMP-LFB amplification conditions, including the target DNA concentration, reaction temperature and time, were optimized. The sensitivity and specificity of the m-LAMP-LFB method were tested in the current study, and the multiple-LAMP-LFB technology was applied to detect the MSSA and MRSA strains from clinical samples.

**Results:**

The *S. aureus-* and MRSA-specific primers based on the *femA* and *mecA* genes allowed the multiple-LAMP technology to detect *S. aureus* and MRSA, respectively. The multiple-LAMP conditions were optimized at 63 °C for 40 min. The full process, including genomic DNA template preparation, LAMP, and product identification, could be achieved in 80 min. The limit of detection (LoD) of the multiple-LAMP assay for *femA* and *mecA* detection was 100 fg of genomic DNA template per reaction. The specificity of m-LAMP-LFB detection was 100 %, and no cross-reactions to non-*S. aureus* strains were observed.

**Conclusion:**

The multiple-LAMP-LFB technique developed in the current study is a reliable, simple, rapid, specific and sensitive method to identify MSSA and MRSA infections for appropriate antibiotic therapy.

## Background

*Staphylococcus aureus*, a gram-positive coccoid bacterium, is a common human pathogen that has the ability to cause a wide array of severe hospital and community-acquired infections, such as pneumonia, bacteremia, sepsis and toxic shock syndrome [1–3]. *S. aureus* infections have been typically treated with methicillin (a semisynthetic antibiotic), which was developed and applied to clinical practice in the late 1950s [4]. Unfortunately, a methicillin-resistant *S. aureus* (MRSA) strain was found among clinical isolates from patients hospitalized in 1960. By the 1980s, MRSA strains were globally epidemic in both community and healthcare settings [5–7]. According to a 2014 World Health Organization (WHO) report, MRSA was listed as one of the seven pathogens of international concern and has been associated with a high number of mortality and mortality [8, 9]. Thus, developing a reliable and rapid method of detection for the accurate differentiation of methicillin-susceptible *S. aureus* (MSSA) and MRSA isolates is necessary for the follow-up treatment and management of patients.

The traditional methods for the detection of MSSA and MRSA is based on cultivation [10] and include colony morphology, biochemical measurements and microdilution drug resistance testing; however, these methods are time-consuming and laborious. These methods require more than 2 days to identify MSSA or MRSA isolates. For this reason, the optimal treatment period is missed for many patients, or antibiotics are abused, resulting in multidrug resistance [11–13]. In recent decades, many molecular methods, such as polymerase chain reaction (PCR), multiplex PCR and real-time PCR, have been applied for detecting MSSA and MRSA isolates [10, 14, 15]. However, PCR-based methods require special experimental instruments and skilled personnel that may not be readily available in many resource-poor settings. Therefore, a cost-effective, simple, reliable, rapid, sensitive, and specific assay for the identification of MSSA and MRSA should be developed to improve treatment and prevent the spread and outbreak of these infections.

To overcome the drawbacks of PCR-based detection, a wide variety of isothermal amplification-based methods have been developed for use in molecular identification. Loop-mediated isothermal amplification (LAMP), as a reliable, low-cost, sensitive and rapid assay, has been widely applied to detect many bacterial pathogens, including *Streptococcus pneumoniae*, *Salmonella* and *Brucella* [16–18]. Unfortunately, the use of the multiplex LAMP (m-LAMP) method to distinguish MRSA from MSSA species has not been reported thus far. LAMP products have been analyzed by various methods, including agarose gel electrophoresis, visual inspection of color changes and turbidimetry changes [19–22]. However, these detection techniques are not specific for target genes and are likely to cause false positive results. To overcome this defect, a target-specific, visual and simple nanoparticle-based lateral flow biosensor (LFB) detection method was successfully designed and applied to analyze m-LAMP products in the current study. Hence, the multiplex-LAMP technique linked to a LFB detector (m-LAMP-LFB) was developed for the reliable, simple, specific, sensitive and visual identification of MSSA and MRSA strains by targeting the *femA* and *mecA* genes, respectively. *femA* is a *S. aureus*-specific gene that appears to be uniquely present in *S. aureus* as it shows no homology with other microbial genomes at GenBank by BLAST searches. *mecA* is an MRSA-specific gene that encodes penicillin-binding protein 2a. The optimal amplification conditions and feasibility of the m-LAMP-LFB assay were confirmed with pure cultures and clinical samples.

## Materials and methods

### Materials instruments

Bacterial genomic DNA extraction kits (QIAamp DNA minikits; Qiagen, Hilden, Germany) were purchased from Qiagen (Beijing, China). Universal isothermal amplification kits, a colorimetric indicator (malachite green, MG), and biotin-14-dCTP were obtained from Bei-Jing HaiTaiZhengYuan. Co., Ltd. (Beijing, China). The LFB materials, including the backing card, sample pad, absorbent pad, conjugate pad and nitrocellulose membrane (NC), were purchased from Jie-Yi Biotechnology. Co., Ltd. (Shanghai, China). Anti-FITC (rabbit anti-fluorescein antibody) and biotin-BSA (biotinylated bovine serum albumin) were purchased from Abcam. Co., Ltd. (Shanghai, China). Dye (Crimson red) streptavidin-coated polymer nanoparticles (129 nm, 10 mg ml^− 1^; 100 mM borate, pH 8.5, with 0.1% BSA, 0.05% Tween 20 and 10 mM EDTA) were purchased from Bangs Laboratories, Inc. (Indiana, USA).

### Design of LAMP detection primers

**Fig. 1:**
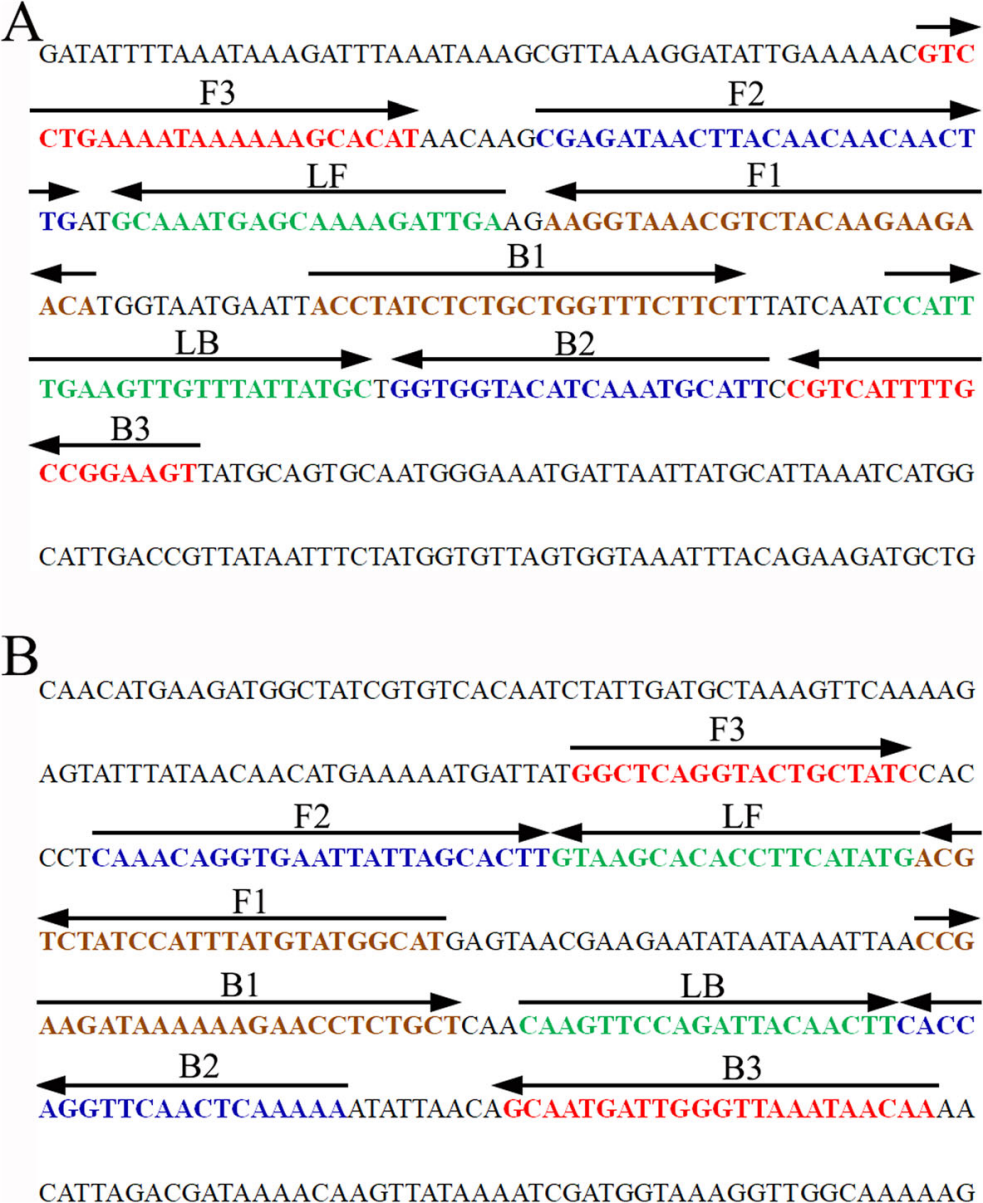
Sequence and location of the *femA* (**a**) and *mecA* (**b**) genes used to design m-LAMP primers. The nucleotide sequences of the sense strand of the *femA* and *mecA* genes are shown in the diagram. Right arrows and left arrows indicate sense and complementary sequences, respectively, which were used in the current study

Based on the reaction mechanism of LAMP, two sets of specific primers were designed according to the target genes *fem*A (GenBank Accession No. NC 007795) and *mecA* (GenBank Accession No. X52593) to detect *S. aureus* and MRSA, respectively. The primers were designed with Primer Explorer V4 (http://primerexplorer.jp/e/; Eiken Chemical Co., Ltd., Tokyo, Japan) online primer design software and checked with the basic local alignment search tool (BLAST). The primer positions are shown in Fig. 1, and the primer sequences and modifications are shown in Table 1. All of the primers were synthesized by TsingKe Biotech Co., Ltd. (Beijing, China) with HPLC purification grade.

**Table 1:** The primers used in the present study

Primer name	Sequence and modifications	Length	Gene
F3	5′-GTCCTGAAAATAAAAAAGCACAT-3′	23 nt	*femA*
B3	5′-ACTTCCGGCAAAATGACG-3′	18 nt	
FIP	5′-TGTTCTTCTTGTAGACGTTTACCTT-CGAGATAACTTACAACAACAACTTG-3′	50 mer	
FIP*	5′-Dig- TGTTCTTCTTGTAGACGTTTACCTT-CGAGATAACTTACAACAACAACTTG −3′	50 mer	
BIP	5′-ACCTATCTCTGCTGGTTTCTTCT-AATGCATTTGATGTACCACC-3′	43 nt	
LF	5′-TCAATCTTTTGCTCATTTGC-3′	20 nt	
LF*	5′-Biotin-TCAATCTTTTGCTCATTTGC-3′	20 nt	
LB	5′-CCATTTGAAGTTGTTTATTATGC-3′	23 nt	
F3	5′-GGCTCAGGTACTGCTATC-3′	18 nt	*mecA*
B3	5′-TTGTTATTTAACCCAATCATTGC-3′	23 nt	
FIP	5′-ATGCCATACATAAATGGATAGACGT-CAAACAGGTGAATTATTAGCACTT-3′	49 nt	
FIP*	5′-FAM-ATGCCATACATAAATGGATAGACGT-CAAACAGGTGAATTATTAGCACTT-3′	49 nt	
BIP	5′-CCGAAGATAAAAAAGAACCTCTGCT-TTTTTGAGTTGAACCTGGTG-3′	45 nt	
LF	5′-CATATGAAGGTGTGCTTAC-3′	19 nt	
LF*	5′-Biotin-CATATGAAGGTGTGCTTAC-3′	19 nt	
LB	5′-CAAGTTCCAGATTACAACTT-3′	20 nt	

Note: *femA*-FIP*, 5′-labeled with Dig when used in LAMP-LFB assay; *femA*-LF*, 5′-labeled with biotin when used in LAMP-LFB assay

*mecA*-FIP*, 5′-labeled with FAM when used in the LAMP-LFB assay; *mecA*-LF*, 5′-labeled with biotin when used in the LAMP-LFB assay;

*Abbreviations*: *Dig* digoxigenin, *FAM* carboxyfluorescein, *nt* nucleotide, *mer* monomeric unit

### Bacterial strains and genomic DNA template preparation

In the current study, a total of 49 strains, including the *S. aureus* reference strain (ATCC 25923), 11 isolated MSSA strains, the methicillin resistant *S. aureus* reference strain (ATCC 43300), 16 MRSA isolated strains, and 20 non-*S. aureus* strains, were used for m-LAMP-LFB detection (Table 2). Genomic DNA templates were obtained using DNA extraction kits in accordance with the manufacturer’s instructions, and the concentration and purity were identified with a Nano drop ND-2000 (Beijing, China) at A260/280. The DNA templates were stored at − 20 °C before use. The genomic DNA of *S. aureus* (ATCC 25923) and MRSA (ATCC 43300) were serially diluted to concentrations ranging from 10 ng/μL to 100 ag/μL (10 ng/μL, 10 pg/μL, 1 pg/μL, 100 fg/μL, 10 fg/μL, 1 fg/μL, and 100 ag/μL), which were used to optimize the reaction temperature, reaction time, and sensitivity.

**Table 2:** Bacterial strains used in the current study

No.	Bacteria	Strain no. (source of strains)^a^	No. of strains	m-LAMP-LFB result^b^	
				*femA*	*mecA*
1	*S. aureus* (MSSA)	ATCC 25923	1	P	N
2	*S. aureus* (MSSA)	Isolated strains (2nd GZUTCM)	11	P	N
3	*S. aureus* (MRSA)	ATCC 43300	1	P	P
4	*S. aureus* (MRSA)	Isolated strains (2nd GZUTCM)	17	P	P
5	*Enterococcus faecalis*	ATCC 29212	1	N	N
6	*Pseudomonas aeruginosa*	ATCC 27853	1	N	N
7	*Shigella flexneri*	Isolated strains (2nd GZUTCM)	1	N	N
8	*Listeria monocytogenes*	Isolated strains (2nd GZUTCM)	1	N	N
9	*Mycobacterium tuberculosis*	Isolated strains (GZCDC)	1	N	N
10	*Acinetobacter baumannii*	Isolated strains (2nd GZUTCM)	1	N	N
11	*Bacillus cereus*	Isolated strains (GZCDC)	1	N	N
12	*Vibrio parahaemolyticus*	Isolated strains (GZCDC)	1	N	N
13	*Leptospira interrogans*	Isolated strains (GZCDC)	1	N	N
14	Enterohemorrhagic *Escherichia coli*	Isolated strains (2nd GZUTCM)	1	N	N
15	Enteroaggregative *Escherichia coli*	Isolated strains (2nd GZUTCM)	1	N	N
16	*Streptococcus pneumoniae*	Isolated strains (2nd GZUTCM)	1	N	N
17	*Staphylococcus saprophyticus*	Isolated strains (GZCDC)	1	N	N
18	Enterotoxigenic *Escherichia coli*	Isolated strains (2nd GZUTCM)	1	N	N
19	Invasive *Escherichia coli*	Isolated strains (2nd GZUTCM)	1	N	N
20	*Haemophilus parainfluenzae*	Isolated strains (2nd GZUTCM)	1	N	N
21	*Shigella boydii*	Isolated strains (2nd GZUTCM)	1	N	N
22	Enteropathogenic *Escherichia coli*	Isolated strains (GZCDC)	1	N	N
23	*Klebsiella pneumoniae*	Isolated strains (2nd GZUTCM)	1	N	N
24	*Bordetella parapertussis*	Isolated strains (GZCDC)	1	N	N

^a^*ATCC* American Type Culture Collection, *2nd GZUTCM* Second Affiliated Hospital, Guizhou University of Traditional Chinese Medicine, *GZCDC* Guizhou Provincial Center for Disease Control and Prevention

^b^*P* Positive, *N* Negative

### Gold nanoparticle-based lateral flow biosensor preparation

The LFB platform was prepared according to a previous report [23]. Briefly, the LFB contained four components: an absorbent pad, NC membrane, sample pad, and conjugate pad. The components were assembled orderly on a backing card. The capture reagents, including anti-Dig, anti-FAM, and biotin-BSA, were immobilized by physical adsorption on the reaction regions. Then, anti-Dig was immobilized at test line 1 (TL1) (MSSA), and anti-FAM was immobilized at test line 2 (TL2) (MRSA), while biotin-BSA was immobilized at the control line (CL); each line was separated by 5 mm. SA-PNPs (dye streptavidin-coated polymer nanoparticles) were gathered on the conjugate pad. The prepared biosensors were preserved in a plastic box with a desiccant gel at room temperature before use.

### The standard LAMP reaction

The single LAMP reactions for MSSA or MRSA were performed in 25 μl reaction systems as previously described [18]. Briefly, 0.4 μM each outer primer, F3 and B3, 0.8 μM each loop primer, LF* and LB, 1.6 μM each inner primer, FIP* and BIP, 0.4 mM biotin-14-dCTP, 1 μl (8 U) of Bst DNA polymerase, 12.5 μl of 2× reaction buffer, and 1 μl of DNA template were added to a tube. The mixtures were heated at 64 °C for 1 h. Genomic DNA from non-*S. aureus* strains, including *Pseudomonas aeruginosa* and *Enterococcus faecalis*, was used as a negative control (NC), and double distilled water (DW) was used as the template in the blank control (BC).

The m-LAMP reaction was performed in a one-step reaction in a 25 μl reaction system containing 12.5 μl of 2× reaction buffer; 0.2 μM each outer primer, *fem*A-F3, *fem*A-B3, *mec*A-F3 and *mec*A-B3; 0.4 μM each loop primer, *fem*A-LF*, *fem*A-LB, *mec*A-LF* and *mec*A-LB; 0.8 μM each inner primer, *fem*A-FIP*, *fem*A-BIP, *mec*A-FIP* and *mec*A-BIP; 0.4 mM biotin-14-dCTP; 1 μl (8 U) of Bst DNA polymerase; and 1 μl of DNA template. The reaction conditions were as described above.

### Detection of LAMP products

Colorimetric indicator (malachite green, MG) and lateral flow biosensor (LFB) methods were applied for the determination and verification of the *fem*A*-*LAMP, *mec*A-LAMP, and m-LAMP products. For the products amplified effectively, the color changed from colorless to light green in the MG assay. However, the color of the negative and blank controls remained colorless. The strategy of visualization of LAMP products with LFB was as previously described [24].

### Temperature optimization of the LAMP assays

The effect of temperature on each LAMP reaction (*fem*A*-*LAMP and *mec*A-LAMP) was tested during the amplification stage. Reaction temperatures ranging from 60 to 67 °C with 1 °C intervals were tested. Reaction mixtures with 1 μL of genomic DNA from *P. aeruginosa* and *E. faecalis* were used as negative controls (NCs), and 1 μL of distilled water (DW) was used as a blank control. The LAMP amplicons were analyzed by examining the turbidity of the products. The curves of the DNA concentrations of each amplified product are shown in the graph. Turbidity > 0.1 was considered positive.

### Analytical sensitivity of LAMP-LFB assays

The sensitivity of each LAMP-LFB reaction (*fem*A*-*LAMP-LFB, *mec*A-LAMP-LFB, and m-LAMP-LFB) was determined using 1 μl of the serial dilution (10 ng, 10 pg, 1 pg, 100 fg, 10 fg, 1 fg and 100 ag per microliter) of the extracted genomic DNA. The LAMP-LFB reactions were carried out as described above, and the results were tested using a colorimetric indicator (MG) and LFB. The limit of detection (LoD) of single and multiplex reactions was verified as the last dilution of each positive test. Three replicates were tested for each dilution.

### Optimization of the amplification time for the multiplex-LAMP-LFB assay

To optimize the reaction time of m-LAMP-LFB, four amplification times (20, 30, 40 and 50 min) were evaluated. The LAMP-LFB reactions were carried out as described above, and the results were tested using LFB. Each amplification time was tested at least three times.

### Specificity analysis of m-LAMP-LFB detection

To determine the specificity of the m-LAMP-LFB assay, genomic DNA (at least 10 ng per microliter) from 12 MSSA strains, 17 MRSA strains, and 20 non-*S. aureus* strains (Table 2) was used for m-LAMP, and all of the results were tested using the LFB method. All examinations were confirmed at least three times.

### Application of the m-LAMP-LFB method to analyze the clinical samples

To verify the applicability of the m-LAMP-LFB method for detecting *S. aureus* and identifying MRSA strains, a total of 63 whole blood samples, which were suspected of *S. aureus* infection, were collected from the Second Affiliated Hospital of Guizhou University of Traditional Chinese Medicine. The clinical samples were detected for MSSA and MRSA using traditional culture, PCR, and m-LAMP-LFB methods. Traditional culture methods included blood culture, colony morphology, Gram staining, biochemical identification, and methicillin susceptibility testing. PCR diagnosis was carried out using *S. aureus-* and MRSA-specific primers targeting the *femA* and *mecA* genes, respectively. The m-LAMP-LFB detection was performed as described above.

## Results

### Verification and analysis of *femA*- and *mecA-*LAMP products

**Fig. 2:**
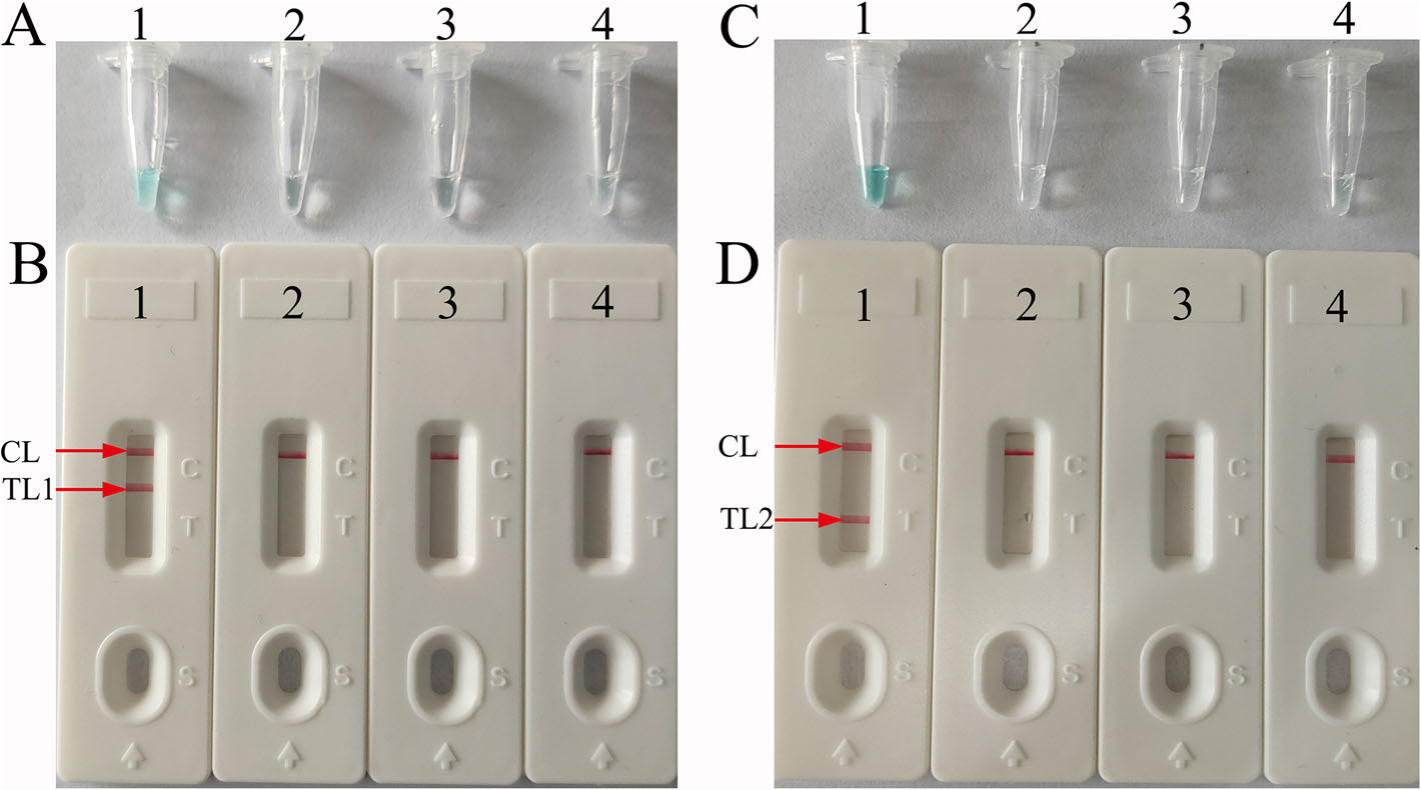
Detection and verification of *femA*-and *mecA*-LAMP products. **a**, **b** Color and lateral flow biosensor detection of *femA*-LAMP products. Tube A1/Biosensor B1, positive amplification of the *femA* gene (*S. aureus*, ATCC25923); Tube A2 /Biosensor B2, negative amplification (*Pseudomonas aeruginosa*); Tube A3/Biosensor B3, negative amplification (*Enterococcus faecalis*); Tube 4/Biosensor 4, blank control (DW). **c**, **d** Color and lateral flow biosensor detection of *mecA*-LAMP products. Tube A1/Biosensor B1, positive amplification of the *mecA* gene (methicillin-resistant *S. aureus*, ATCC43300); Tube A2/Biosensor B2, negative amplification (*Pseudomonas aeruginosa*); Tube A3/Biosensor B3, negative amplification (*Enterococcus faecalis*); Tube 4/Biosensor 4, blank control (DW)

To confirm the amplification with the two sets of LAMP primers, the *femA*-, *mecA*-, and m-LAMP mixtures were incubated at a constant temperature of 63 °C for 1 h. Then, the *femA*-, *mecA*-, and m-LAMP products were analyzed with colorimetric indicator (MG) and lateral flow biosensor (LFB) methods, respectively. The color of the positive results in the *femA*-, *mecA*-, and m-LAMP reactions changed from colorlessness to bright green, while the negative and blank control reactions remained colorless (Fig. 2a, c). LFB was used for further confirmation of *femA*-, *mecA*-, and m-LAMP. For *femA*-LAMP detection, two crimson red bands (CL and TL1) appeared, indicating positive results, and CL and TL2 were visible for *mecA*-LAMP, indicating successful amplification, while the negative and blank controls only appeared as a crimson red line (CL) in the biosensor (Fig. 2b, d). Therefore, the results suggested that the two sets of LAMP primers for *femA* and *mecA* detection were valid for the development of the m-LAMP techniques.

### Optimal amplification temperature for *femA*- and *mecA*-LAMP

**Fig. 3:**
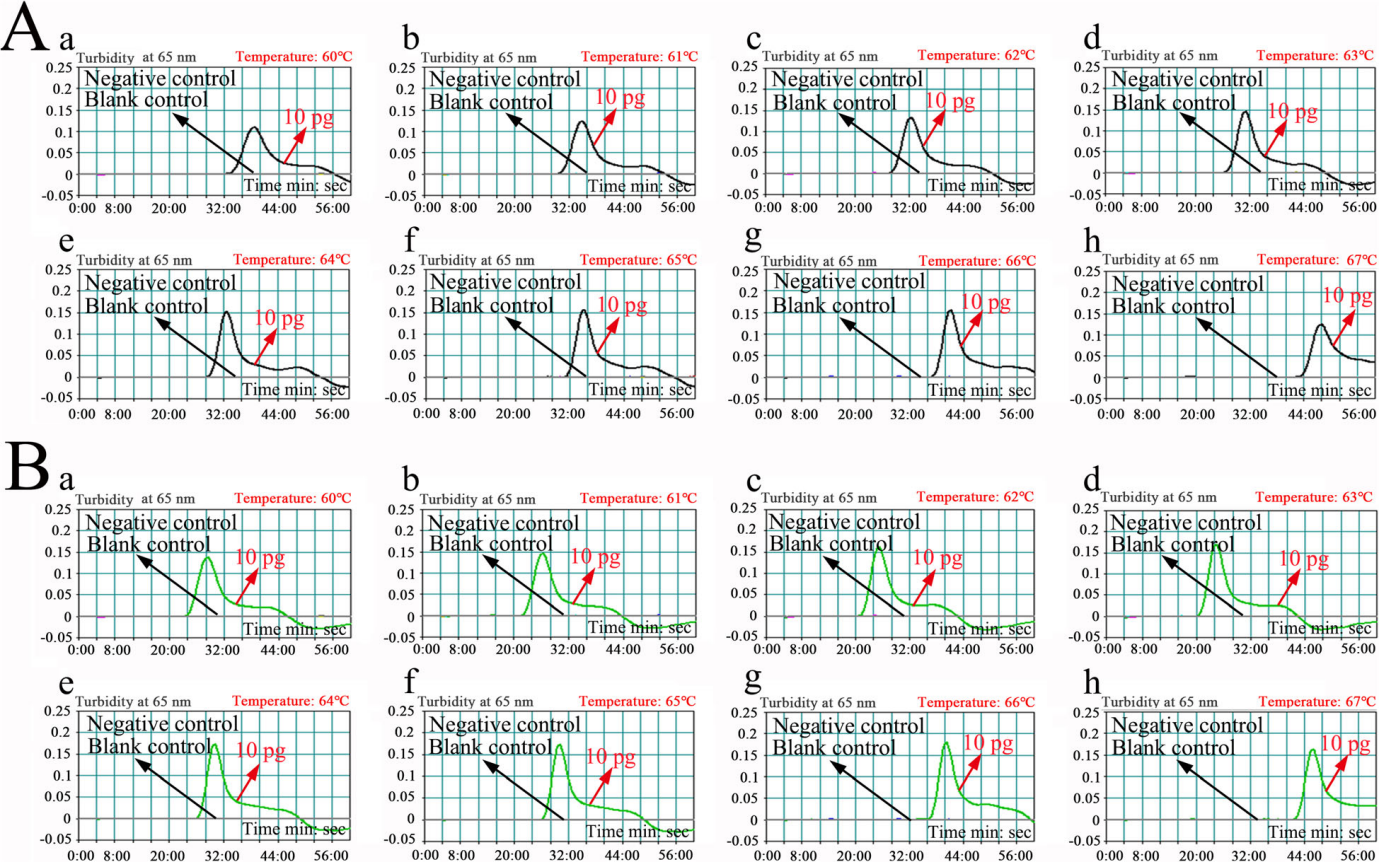
Optimization of amplification temperature for the *femA*-LAMP (**a**) and *mecA*-LAMP (**b**) primer sets. LAMP for the detection of *femA* (**a**) and *mecA* (**b**) was monitored through real-time turbidity, and the corresponding curves of DNA concentrations are displayed in the graphs. The threshold value was 0.1, and turbidity> 0.1 was considered positive. Mixtures with 10 pg of genomic DNA from *P. aeruginosa* (ATCC 27853) *and E. faecalis* (ATCC 29212) were used as negative controls (NCs), and 1 μl of double distilled water (DW) was used as a blank control (BC). Eight kinetic graphs were obtained at different temperatures (60–67 °C, 1 °C intervals) with 10 pg of target genomic DNA per reaction. **a**, The graphs from **d** (63 °C) to **g** (66 °C) showed robust amplification; **b,** the graphs from **b** (61 °C) to **h** (67 °C) showing robust amplification

The reaction temperature is crucial for LAMP. In this study, the reaction temperature of *femA*- and *mecA*-LAMP was tested at different temperatures (from 60 to 67 °C with 1 °C intervals) with genomic template (10 pg/μl) extracted from purified cultures (ATCC 43300). The LAMP protocol was performed as described above, and the *femA*- and *mecA*-LAMP reactions were monitored by means of real-time turbidity measurements. Kinetic graphs were recorded at all temperatures. The results showed that *femA*-LAMP was amplified faster in the temperature range from 63 to 66 °C, and *mecA*-LAMP was amplified faster at 61 to 67 °C (Fig. 3). Hence, the amplification temperature of 63 °C was considered the optimal temperature for the rest of the m-LAMP reactions in the present study.

### Sensitivity of *femA*- and *mecA*-LAMP detection

**Fig. 4:**
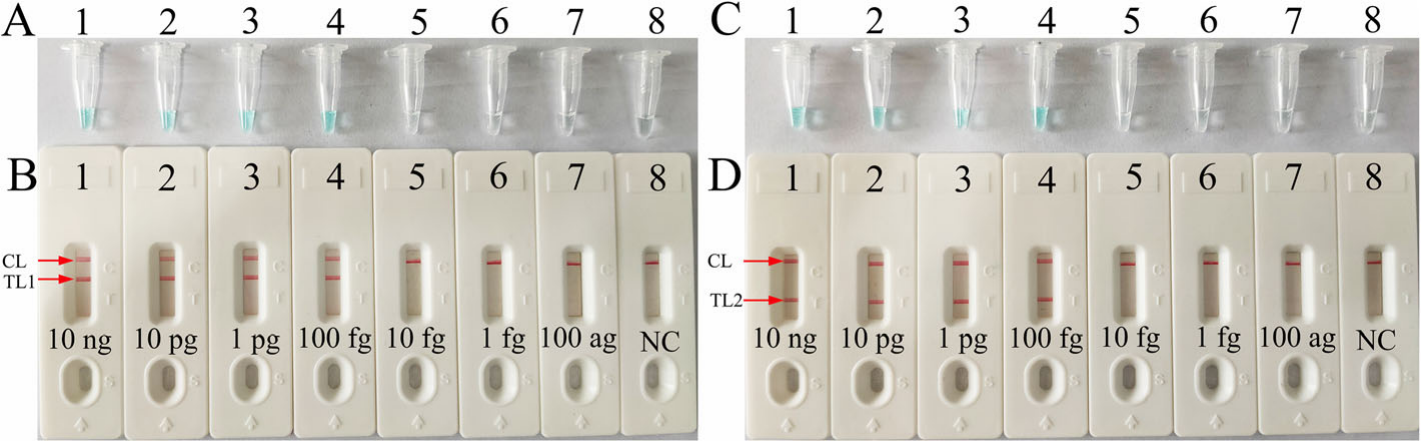
Sensitivity analysis of *femA*-LAMP (**a**, **b**) and *mecA*-LAMP (**c**, **d**) detection with serial dilutions of genomic DNA extracted from MSSA and MRSA strains, respectively Two detection methods involving a colorimetric indictor (MG; **a**, **c**) or lateral flow biosensor (**b**, **d**) were used to analyze the amplification products. The genomic DNA was serially diluted (10 ng, 10 pg, 1 pg, 100 fg, 10 fg, 1 fg and 100 ag per microliter) and subjected to standard LAMP. Tubes A1-A7 (Biosensors B1-B7), *S. aureus* (ATCC25923) genomic templates (10 ng-100 ag); Tube A8 (Biosensor B8), negative control (DW). The LoD of *femA*-LAMP detection was 100 fg of genomic template per reaction. Tubes C1-C7 (Biosensors D1-D7), methicillin-resistant *S. aureus* (ATCC43300) genomic templates (10 ng-100 ag); Tube C8 (Biosensor D8), blank control (DW). The LoD of *mecA*-LAMP detection was 100 fg of genomic template per reaction.

The sensitivity of *femA*- and *mecA*-LAMP detection was evaluated with serially diluted genomic DNA at concentrations ranging from 10 ng to 100 ag per microliter. The LAMP amplicons were analyzed by visual inspection with MG reagents and lateral flow biosensors. The CL and TL1 lines appeared on the biosensor, showing positive results for the *femA-*LAMP assay, and two crimson lines (CL and TL2) were observed on the biosensor, indicating positive results for *mecA-*LAMP detection. For the negative controls, only the CL line appeared on the biosensor, indicating negative results. The results showed that the LoD of *mecA*-LAMP was 100 fg per reaction, which was the same as the LoD of the *femA*-LAMP assay (Fig. 4).

### Sensitivity of m-LAMP detection

**Fig. 5:**
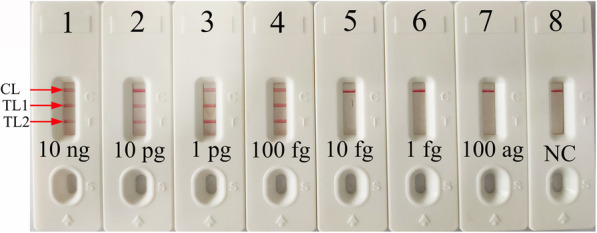
Sensitivity analysis of m-LAMP detection with serial dilutions of genomic DNA extracted from the MRSA strain. Two sets of primers targeting the *femA* and *mecA* genes were simultaneously added to a reaction vessel, and the LoD of m-LAMP for detecting *S. aureus* and identifying MRSA was analyzed with a lateral flow biosensor. Biosensors 1–8 represent the genomic DNA (methicillin-resistant *S. aureus*, ATCC43300) amounts of 10 ng, 10 pg, 1 pg, 100 fg, 10 fg, 1 fg and 100 ag per reaction and blank control (DW), respectively. The LoD of the m-LAMP assay for *femA* and *mecA* detection was 100 fg of genomic template per reaction

After m-LAMP was performed, the products were directly analyzed using LFB. The CL, TL1, and TL2 bands became crimson on the biosensor, reporting positive results for the *femA* and *mecA* genes. Only the CL line appeared on the biosensor, indicating negative results. The results showed that the LoD of m-LAMP-LFB for simultaneously assaying *femA* and *mecA* genes was also 100 fg of DNA per reaction (Fig. 5), which was consistent with single LAMP detection (Figs. 4 and 5).

### Optimization of amplification time for m-LAMP-LFB detection

**Fig. 6:**
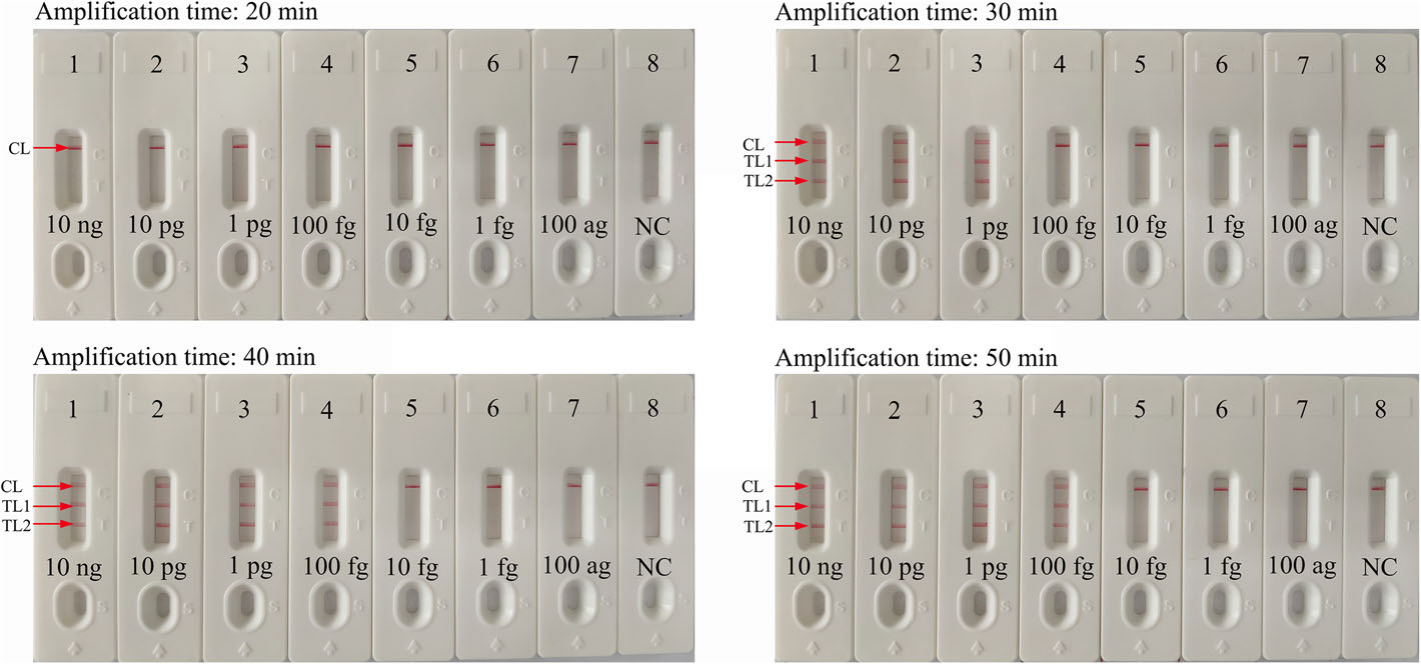
Optimization of the amplification time for m-LAMP detection. Different amplification times (**a**, 20 min; **b**, 30 min; **c**, 40 min; **d**, 50 min) were tested at 63 °C. Biosensors 1, 2, 3, 4, 5, 6, 7, and 8 represent genomic DNA (methicillin-resistant *S. aureus*, ATCC43300) levels of 10 ng, 10 pg, 1 pg, 100 fg, 10 fg, 1 fg and 100 ag target template per reaction and negative control (DW), respectively. The best sensitivity was observed when the amplification lasted for 40 min (**c**)

To obtain an optimal reaction time for m-LAMP, four reaction times (20, 30, 40, and 50 min) were tested at the optimal amplification temperature (63 °C). The results showed that the LoD of the genomic DNA template (100 fg of MRSA per reaction) was detected (displayed TL1, TL2 and CL) when the m-LAMP lasted 40 min (Fig. 6). Hence, a reaction time of 40 min was considered the optimal reaction time for m-LAMP detection. In summary, the whole detection procedure, including target genomic DNA preparation (30 min), m-LAMP (40 min) and analysis of results (2 min), could be completed within 80 min.

### Specificity of the m-LAMP assay

**Fig. 7:**
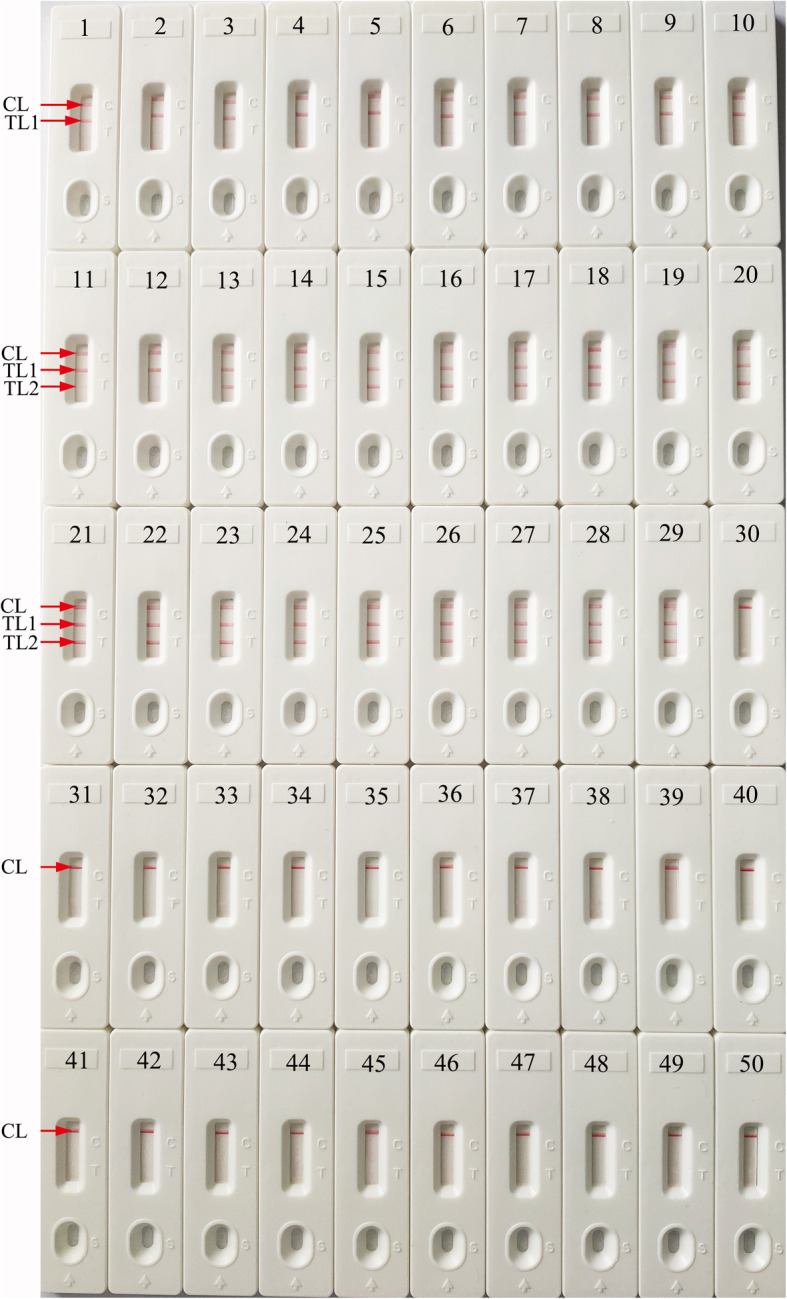
Specificity analysis of m-LAMP-LFB detection using different strains. The m-LAMP reactions were carried out using different genomic DNA as templates, and each of the amplification products was determined by means of the visual LFB method. Biosensor 1, MSSA (ATCC 25923); Biosensors 2–12; eleven isolated strains of MSSA from the Second Affiliated Hospital, Guizhou University of Traditional Chinese Medicine; Biosensor 13, MRSA (ATCC 43300); Biosensors 14–29, sixteen isolated strains of MRSA from the Second Affiliated Hospital, Guizhou University of Traditional Chinese Medicine; Biosensors 30–49, *Enterococcus faecalis*, *Pseudomonas aeruginosa*, *Shigella Flexneri*, *Listeria monocytogenes*, *Mycobacterium tuberculosis*, *Acinetobacter baumannii*, *Bacillus cereus*, *Vibrio parahaemolyticus*, *Leptospira interrogans*, enterohemorrhagic *Escherichia coli*, enteroaggregative *Escherichia coli*, *Streptococcus pneumonia*, *Staphylococcus saprophyticus*, enterotoxigenic *Escherichia coli*, invasive *Escherichia coli*, *Hemophilus parainfluenza*, *Shigella boydii*, enteropathogenic *Escherichia coli*, *Klebsiella pneumonia*, *Bordetella parapertussis*, respectively; biosensor 50, blank control (DW)

The specificity of m-LAMP detection was confirmed with MSSA, MRSA, and non-*S. aureus* isolates (Table 2). The genomic DNA extracted from MSSA and MRSA strains presented positive results. Three crimson lines (TL1, TL2 and CL) were displayed on the LFB, indicating positive results for the MRSA isolates. TL1 and CL appeared on the LFB, indicating positive results for the MSSA isolates. Other non-*S. aureus* strains and the blank control showed negative results (Fig. 7). Hence, the results confirmed that the m-LAMP-LFB method could accurately identify *S. aureus* and differentiate MRSA from all *S. aureus* strains.

### Feasibility of the m-LAMP-LFB method using whole blood samples

To further demonstrate the feasibility of m-LAMP-LFB as a valuable method for the detection of MRSA and MRSA, 63 whole blood samples of suspected *S. aureus*-infected patients were collected from the Second Affiliated Hospital of Guizhou University of Traditional Chinese Medicine and tested by conventional culture-biotechnical methods, PCR detection, and m-LAMP-LFB assays. The results showed that 16 of 63 samples had been verified as MSSA-positive, and 12 of 63 samples had been verified as MRSA-positive through traditional culture techniques. The m-LAMP-LFB assay results were consistent with the traditional cultivation detection results. However, using PCR, only 14 and 9 samples were confirmed as MSSA- and MRSA-positive results, respectively (Table 3). These results suggested that the m-LAMP-LFB assay established in the current study could be used as an advanced tool to detect all *S. aureus* strains and separate MRSA from MSSA.

**Table 3:** Comparison of conventional culture, PCR and m-LAMP-LFB methods to identify MSSA and MRSA in clinical samples

Detection method	Clinical samples (*n* = 63)		
	MSSA-Positive	MRSA-Positive	Negative
Culture	16	12	35
PCR	14	9	40
m-LAMP-FB	16	12	35

## Discussion

*S. aureus*, a ‘Janus-faced’ bacterium, is a commensal species and a pathogenic microorganism [25–27]. It is estimated that 20–30% of adult populations carry *S. aureus* in their nares; although this bacterium is commonly found, it only causes invasive infection when the host immune system is weakened [28–30]. In nosocomial settings, *S. aureus* is widely distributed in the environment, on surgical instrument surfaces, and on implanted medical devices, including prosthetic joints, catheters, and artificial heart valves [11, 31]. After *S. aureus* invades the human body, the pathogen rapidly enters the bloodstream and then diffuses into vital organs, causing pathological injury, such as osteomyelitis, endocarditis, and descending urinary tract infections [28]. Importantly, since methicillin-resistant *S. aureus* has emerged and spread worldwide, *S. aureus* has disrupted both the healthcare setting and community [4, 30], resulting in a huge socioeconomic burden in both developed and developing areas. The detection and identification of MSSA and MRSA is essential in cases of suspected *S. aureus* infections. However, traditional methods, including culture-based techniques, colony morphology, Gram staining, biochemical identification, methicillin susceptibility testing, and PCR-based detection (traditional PCR, multiple PCR and real-time PCR approaches), are time consuming and require expensive instruments. Moreover, the accurate interpretation of the results requires trained experts [14, 32]. Herein, developing a reliable, rapid, low-cost, simple, specific and sensitive detection method to accurately differentiate MSSA and MRSA is essential for disease diagnosis and therapy.

In the current study, m-LAMP-LFB detection targeting the *femA* and *mecA* genes was successfully established to assay all *S. aureus* species and identify MRSA. The sequences of *S. aureus*-LAMP primers were designed using the *femA* gene, which appears to be a unique feature of *S. aureus* and is not found in other *Staphylococcus* species [10]. Moreover, the MRSA-LAMP primers were designed with the *mecA* gene, encoding the low-affinity penicillin-binding protein 2a (PBP2a), which is associated with the methicillin resistance of *S. aureus* species [33]. The specificity of the m-LAMP assay was confirmed with genomic DNA from 12 MSSA, 17 MRSA, and 20 non-*S. aureus* isolates. m-LAMP detection of the *femA* gene identified *S. aureus* with 100% specificity, and the *mecA* gene identified MRSA with 100% specificity (Fig. 7).

In the present study, a nanoparticle-based lateral flow biosensor (LFB) was applied to analyze LAMP products. Although the amplification results could be detected equally with the turbidity and MG methods used in the current study, the LFB was deemed the preferred method for analyzing LAMP products; the LFB provides more visualization and does not require special instruments, regents and processes [34, 35]. In particular, the LFB applied in this study can simultaneously and visually detect two target genes (*femA* and *mecA*) in a single test. Compared with conventional culture and PCR-based methods, the m-LAMP-LFB technique is more sensitive, time-saving and cost-saving. The newly developed m-LAMP-LFB method was able to detect 100 fg of genomic DNA (Figs. 4 and 5). The entire detection process, including template preparation (approximately 30 min), isothermal amplification (40 min) and LFB reading (approximately 2 min), could be accomplished within 80 min. The total cost of one test, including genomic DNA extraction (approximately $1 USD), LAMP reaction (approximately $3.5 USD) and LFB reading (approximately $2 USD), is estimated to be $6.5 USD, which is cheaper than normal PCR-based methods. In addition, the advanced technique can decrease labor costs because performing the LAMP-LFB assay does not require skilled technical personnel. In conclusion, the newly developed m-LAMP-LFB technique in this study is a rapid, reliable and low-cost assay for the identification of MSSA and MRSA, and this technique can save detection time and help determine the optimal treatments for patients in a timely manner. In addition, accurate, timely and low-cost testing can reduce the patient’s financial burden, especially in resource-constrained regions of the world.

## Conclusions

In the current study, a reliable, rapid and simple m-LAMP-LFB technique based on the *femA* and *mecA* genes was successfully developed for assaying *S. aureus* and identifying MRSA. This method could reliably, specifically, sensitively and rapidly detect all *S. aureus* species and identify MRSA isolates in samples. The amplification products were analyzed with LFB, which was objective, rapid, and easily interpretable. Hence, the m-LAMP-LFB assay could be considered a useful method for the reliable and rapid detection of *S. aureus* and identification of MRSA in clinical samples, particularly in resource-constrained regions of the world.

## Data Availability

The datasets used and/or analyzed during the current study are available from the corresponding author on reasonable request.

## Abbreviations

MSSA: Methicillin susceptible *Staphylococcus aureus*
MRSA: Methicillin resistant *Staphylococcus aureus*
LAMP: Loop-mediated isothermal amplification
LFB: Lateral flow biosensor
m-LAMP-LFB: multiplex loop-mediated isothermal amplification linked to a nanoparticle-based lateral flow biosensor
LoD: Limit of detection
WHO: WORLD Health Organization
PCR: Polymerase chain reaction
MG: Malachite green
ATCC: American type culture collection
2nd GZUTCM: Second Affiliated Hospital, Guizhou University of Traditional Chinese Medicine
GZCDC: Guizhou Provincial Center for Disease Control and Prevention
Dig: Digoxigenin
FAM: Carboxyfluorescein
nt: nucleotide
mer: monomeric unit
TL1: Test line 1
TL2: Test line 2
CL: Control line
NC: Negative control
BC: Blank control
DW: Distilled water
PBP2a: Penicillin-binding protein 2a
